# Multi-Scale Computational Models for Electrical Brain Stimulation

**DOI:** 10.3389/fnhum.2017.00515

**Published:** 2017-10-26

**Authors:** Hyeon Seo, Sung C. Jun

**Affiliations:** School of Electrical Engineering and Computer Science, Gwangju Institute of Science and Technology, Gwangju, South Korea

**Keywords:** multi-scale model, electrical brain stimulation, finite element model, volume conductor model, multi-compartmental neuronal model, cortical neuron

## Abstract

Electrical brain stimulation (EBS) is an appealing method to treat neurological disorders. To achieve optimal stimulation effects and a better understanding of the underlying brain mechanisms, neuroscientists have proposed computational modeling studies for a decade. Recently, multi-scale models that combine a volume conductor head model and multi-compartmental models of cortical neurons have been developed to predict stimulation effects on the macroscopic and microscopic levels more precisely. As the need for better computational models continues to increase, we overview here recent multi-scale modeling studies; we focused on approaches that coupled a simplified or high-resolution volume conductor head model and multi-compartmental models of cortical neurons, and constructed realistic fiber models using diffusion tensor imaging (DTI). Further implications for achieving better precision in estimating cellular responses are discussed.

## Introduction

Electrical brain stimulation (EBS) is an intriguing electrotherapy designed to regulate cortical excitability through a regulated current, and is used increasingly to treat various neurological disorders and as an adjunct to medical therapy for depression (Padberg and George, 2009; Nahas et al., 2010); chronic pain (Hanajima et al., 2002; Di Lazzaro et al., 2004; Holsheimer et al., 2007; Lefaucheur et al., 2010); rehabilitation (Brown et al., 2003, 2006; Canavero et al., 2006; Levy et al., 2008); Parkinson’s disease (Canavero et al., 2002; Hanajima et al., 2002; Pagni et al., 2008); essential tremor (Picillo et al., 2015); epilepsy (Nitsche and Paulus, 2009; Canavero, 2014); tinnitus (Tass et al., 2012), and other brain disorders (Canavero, 2009). The stimulation current required to modulate cortical excitability can be delivered via invasive (deep brain stimulation (DBS), subdural, or epidural cortical stimulation) and noninvasive (transcranial magnetic or electrical stimulation) methods, depending upon whether a surgical procedure is required.

In invasive cortical stimulation, electrodes are implanted chronically, either epidurally or subdurally, which allows a brain area to be targeted selectively. Further, it is able to deliver external current to the brain with reduced current loss compared to noninvasive stimulation, because electrodes are implanted under the skull and scalp, both of which have high electrical resistance (Canavero, 2009, 2014). DBS is an invasive approach that has been acknowledged worldwide in the treatment of Parkinson’s disease with electrodes implanted deeply (Deuschl et al., 2006; Adamchic et al., 2014). In contrast, transcranial magnetic stimulation (TMS) and transcranial current stimulation (TCS) with pulsed or direct current are noninvasive techniques. TMS generates a time-varying magnetic field using a coil that induces an electric field and activates neurons to produce action potentials (Barker et al., 1986; Wagner et al., 2007). As TMS can generate strong currents that are able to activate neurons, it has been used not only as a treatment for neurological and psychiatric disorders, but also as a diagnostic tool (Di Lazzaro et al., 1999; Schulz et al., 2013). While TMS is a neurostimulation approach that activates neurons, TCS is a neuromodulatory approach that uses low current (~2 mA) to shift the resting membrane potential (Wagner et al., 2007; Nitsche et al., 2008). Although TCS modulates cortical excitability without inducing action potentials, it has the advantages of low cost, portability and ease of use. Further, the transcranial alternating current stimulation (tACS) technique developed recently is able to modulate oscillatory brain activity directly (Fröhlich and McCormick, 2010; Zaehle et al., 2010; Herrmann et al., 2016).

Brain stimulation may be optimized by tailoring individual stimulation parameters and by targeting the neural networks involved selectively; however, there are many uncertainties with respect to efficient stimulation parameters. Electricity can be used as a predictor of the cortical areas affected, and a computational study using a volume conductor model of a head (head model) is the most cost-effective way to demonstrate the stimulus-induced electrical current flow in the brain with a myriad of possible combinations of stimulus parameters (e.g., electrode position, stimulus amplitude and frequency and electrical and geometrical properties of the head).

**Figure 1:**
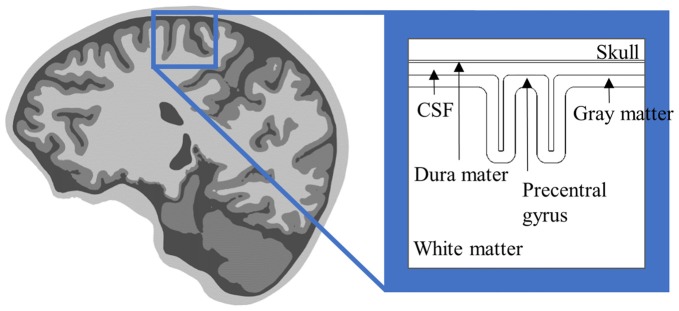
Anterior-posterior cross-section of the extruded slab model of the precentral gyrus. The 3D model is constructed by extruded cross-section. An invasive electrode can be placed on/under the dura mater for epidural/subdural cortical stimulation.

The simplified head model is a concentric sphere composed of three or four layers based on the assumption that the head is a perfect sphere, and is a model used widely for noninvasive brain stimulation (Roth et al., 1991; Thielscher and Kammer, 2002; Datta et al., 2008). Another form of simplified head model for invasive brain stimulation is an extruded slab model that represents a part of the brain, typically the precentral gyrus (Figure 1; Manola et al., 2005, 2007; Wongsarnpigoon and Grill, 2008, 2012; Zwartjes et al., 2012). These models are useful, as they allow direct comparison of various parameters without concomitant complex computations. In recent years, as the importance of brain anatomy has been recognized, some high-resolution head models that reflect geometrical information from magnetic resonance imaging (MRI) have been proposed (Datta et al., 2009; Lee et al., 2012; Edwards et al., 2013; Truong et al., 2013; Windhoff et al., 2013; Kim et al., 2014; Shahid et al., 2014). These models hold promise for realistic electric field calculations that result thereby in more precise estimations of the brain areas affected.

With these head models, we can estimate the degree of activation on the macroscopic level using the stimulus-induced electric field based on a quasi-uniform assumption that neural polarization is proportional to the electric field magnitude (Bikson et al., 2012, 2013). Similarly, the second derivative of the potential, called the activating function, is considered to estimate neural activation by reflecting neural orientations; positive values represent depolarization and negative values indicate hyperpolarization (Rattay, 1989; Manola et al., 2005; Miranda et al., 2007; Silva et al., 2008; Wongsarnpigoon and Grill, 2008). However, the activating function shows simple inverted signs between anodal (when the positive electrode, the anode, is located close to the target area and the reference electrode is situated far away) and cathodal stimulations (when the negative electrode, the cathode, is located close to the target area) because of the linearity of the head model with respect to electric fields. Thus, interpolation using electric fields may be quite trivial, because it ignores inherently complicated features of neurons, such as their structures, electrical properties and functions (Chan and Nicholson, 1986; Bikson et al., 2004; Radman et al., 2009; Seo et al., 2017b).

To unravel the neural mechanics of activation, simulations of neural stimulation have been established to provide the closest approximation (Wu et al., 2016), and a host of neural stimulations have been simulated using Hodgkin and Huxley’s model with modifications (Rattay, 1989; Roth and Basser, 1990; Roth et al., 1991; Nagarajan et al., 1993; Rattay et al., 2003). Simulation of individual neuronal activity provides direct responses that may provide evidence of neurons recruited by brain stimulation (Phillips and Porter, 1962). This requires detailed information about anatomical structure and electrophysiological properties, and thus, morphologically realistic neuronal models are constructed based on extensions of Rall’s (1959) cable model, which may help predict the effects of extracellular electrical stimulation (Herz et al., 2006). With respect to network activity, a large-scale neuronal model with connections has been developed to investigate the effects of neural stimulation on the oscillatory activity patterns and to provide appropriate stimulation parameters. For example, there is a network model of the basal ganglia for closed-loop DBS combined with the desynchronizing delayed feedback approach (Popovych et al., 2017), a model of the motor cortex for reproduction of indirect responses for TMS (Esser et al., 2005; Rusu et al., 2014), and a thalamocortical network that exhibits gamma oscillation, sleep spindles and epileptogenic bursts (Traub et al., 2005). In addition, a neural field model can reproduce the human electroencephalogram (EEG) signals by taking into account cortico-cortical fibers as a major factor (Nunez and Cutillo, 1995).

To simulate the effects of anatomical information on neuronal activation more precisely, numerical approaches that use cortical neuronal models that incorporate electrical and chemical information of biologically realistic neurons have been conducted, and the electricity calculated with head models is used as input to cortical neurons that simulate neural responses. These integrations between neuronal and head models are referred to as multi-scale computational models, and they provide potential neural targets by brain stimulation. Multi-scale computational models remain an area of ongoing research because of the technical difficulty of combining two models. Therefore, rather than constructing complex cortical networks, individual neurons or simple synaptic connections that generate electrical activity are modeled first. Table 1 provides an overview of the literature that summarizes the modeling approaches used to simulate neural responses; these are discussed later in the text.

**Table 1:** Literature survey: modeling approaches for computational estimation of neural responses.

Studies	Type of stimulation	Head model	Neuron type	Compartments of neuron	Goals of investigation
Manola et al. (2005)	ECS	Extruded slab model	Afferent/Efferent fibers	Axon	Explore electric potential field, activating functions, and response of simple fiber models
Manola et al. (2007)	ECS	Extruded slab model	Afferent/Efferent fibers	A single apical dendrite, soma and axon	Evaluate the effect of anodal and cathodal stimulation using pyramidal neurons, including a soma and dendrites
Wongsarnpigoon and Grill (2012)	ECS	Extruded slab model	Layer 3/layer 5 pyramidal neurons and thalamocortical axon	Dendrites, soma and axon	Investigate neuronal activation by varying electrode positions, geometries and polarities
Seo et al. (2016a)	SuCS	Extruded slab model/ anatomically realistic head model	Layer 3/layer 5 pyramidal neurons	Dendrites, soma and axon	Compare simulated responses of cortical neurons between the simplified and full-resolution head models
Zwartjes et al. (2012)	ECS/SuCS	Extruded slab model	Basket neuron, and intratelecenphalic/pyramidal tract neurons	Axon	Determine selective targeting stimulation protocols
Silva et al. (2008)	TMS	Extruded slab model	Fibers aligned either Perpendicular or tangential to the cortical surface	N/A	Investigate the effect of the heterogeneity of electrical properties of the head model on the spatial distribution of the electric field and field gradient
Salvador et al. (2011)	TMS	Extruded slab model	Pyramidal neurons, inter neurons, and association fibers	A single apical dendrite, soma, and axon	Investigate neuronal responses with different current directions and pulse waveforms
Kamitani et al. (2001)	TMS	N/A (RLC-circuit)	Layer 5 pyramidal neurons	Dendrites, soma, and axon	Simulate responses of realistic pyramidal neurons induced by a single magnetic pulse
Pashut et al. (2011)	TMS	N/A (RLC-circuit)	Straight axon, and layer 5 pyramidal neurons	Dendrites, soma and axon	Describe the effect of magnetic stimulation on cortical neurons with arbitrary morphologies
Rahman et al. (2013)	tDCS	N/A (Uniform EF)	Layer 3/layer 5 pyramidal neurons	Dendrites, soma and axon	Address which compartments are associated with excitatory synaptic efficacy
Seo et al. (2015)	SuCS	Anatomically realistic head model	Layer 3/layer 5 pyramidal neurons	Dendrites, soma and axon	Investigate the influence of anisotropic white matter conductivity on the activation of cortical neurons
Seo et al. (2017b)	tDCS + transcranial channel	Anatomically realistic head model	Layer 3/layer 5 pyramidal neurons	Dendrites, soma and axon	Determine whether inclusion of a transcranial channel performs effectively with respect to focalized neuromodulation
Goodwin and Butson (2015)	TMS	Anatomically realistic head model	Layer 3 pyramidal neurons	Dendrites, soma and axon	Predict activated neural tissue by changing coil orientation and waveform
Seo et al. (2016b)	TMS	Anatomically realistic head model	Layer 3/layer 5 pyramidal neurons	Dendrites, soma and axon	Simulate neural activation patterns for different coil orientations
Opitz et al. (2011)	TMS	Anatomically realistic head model	Tractography-based pyramidal tracts and U-pathway	N/A	Highlight the importance of realistic field calculations and demonstrate the necessity of using realistic nerve models
Nummenmaa et al. (2014)	TMS	Anatomically realistic head model	Tractography-based pyramidal tracts	N/A	Determine optimal position and orientation of the TMS coil to maximize neural activation
Shahid et al. (2014)	tDCS	Anatomically realistic head model	Tractography-based fiber bundles	N/A	Consider complexities that influence clinical decisions and provide neural activities to understand the role of fiber pathways
De Geeter et al. (2015)	TMS	Anatomically realistic head model	Tractography-based fiber bundles	N/A	Introduce flexible and personalized modeling by implementing realistic 3D neural trajectories
De Geeter et al. (2016)	TMS	Anatomically realistic head model	Tractography-based fiber bundles	A single apical dendrite, soma, and axon	Investigate the spatial distribution of the membrane polarizations along fiber tracts and their temporal dynamics

*ECS, epidural cortical stimulation; SuCS, subdural cortical stimulation; tDCS, transcranial direct current stimulation*.

## Modeling Activation of Cortical Neurons Produced by Invasive Brain Stimulation

**Figure 2:**
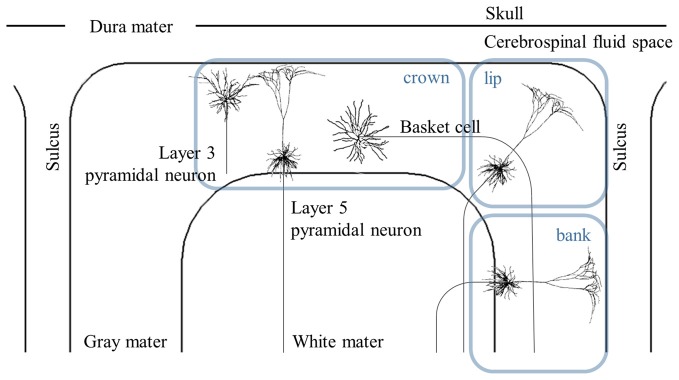
Schematic view of distribution of the cortical neurons. The cross-sectional view of the simplified head model illustrates layer 3 and 5 pyramidal neurons (PN), and basket cells. The cortex can be represented by the crown, which is the top of the gyrus, the lip and the bank along the sulcus. Note that the axons of layer 5 PN in the lip and bank bend after they cross the boundary between the gray matter and white matter. Modified from Canavero (2014) with permission from De Gruyter Open.

Multi-scale computational models are constructed by coupling the head model with multi-compartmental neuron models. As Figure 2 shows, rather than modeling all types of neurons, pyramidal neurons (PN), inter neurons, and basket cells that may be involved in the clinical effects of brain stimulation typically are modeled within the cortex.

Multi-scale models that combine the simplified head and neuronal models are advantageous, in that it is easy to couple neurons with the head model in a straightforward manner because of its typical and simple geometry. As invasive stimulation may stimulate a relatively small region of the brain, simplified head models are used widely to simulate invasive brain stimulation. However, these models may introduce modeling error that results in inaccurate estimation of neuronal activation, and the generalized dimensions of the simplified head model cannot reflect individual anatomical variations and abnormalities in susceptible populations, such as skull defects and lesions (Bikson et al., 2012). Thus, modeling relevant anatomy precisely may help calculate not only the precise pattern of the stimulus-induced electric field, but also neuronal excitability (for multi-scale modeling using the precise head model, see “High-Resolution Models for Brain Stimulation” section).

The neuronal models in this study consisted of a series of compartments connected by resistors. Thus, to estimate the responses of cortical neurons computationally, rather than combining neuronal models directly with the head model, a series of center points comprised of compartments of neuronal models were positioned virtually. Then, electric potentials were computed at the center point of each compartment of neurons using the head model and applied to neuronal models by extracellular stimulation. The membrane polarizations of neuronal models induced by an external field are described by the cable equation (Roth and Basser, 1990; Roth, 1994). One of the practical platforms for biologically realistic neuronal models is NEURON (Hines and Carnevale, 1997).

For invasive cortical stimulation, Manola et al. (2005) provided an initial comparative assumption about the responses of myelinated nerve fibers using a small number of neuronal models. Afferent fibers that were oriented parallel to the cortical surface, and efferent fibers with the same orientations as layer 5 PN in Figure 2 were modeled. They found that efferent fibers in the crown, which lay directly beneath the epidural electrode, were depolarized by anodal stimulation and hyperpolarized by cathodal stimulation, which was consistent with observations using the activating function. In addition, when simple fiber models were extended by including soma and simplified dendrites (Manola et al., 2007), reduced excitation thresholds (minimum stimulus amplitude to evoked action potentials) generally were induced compared to the responses of simple fiber models.

**Figure 3:**
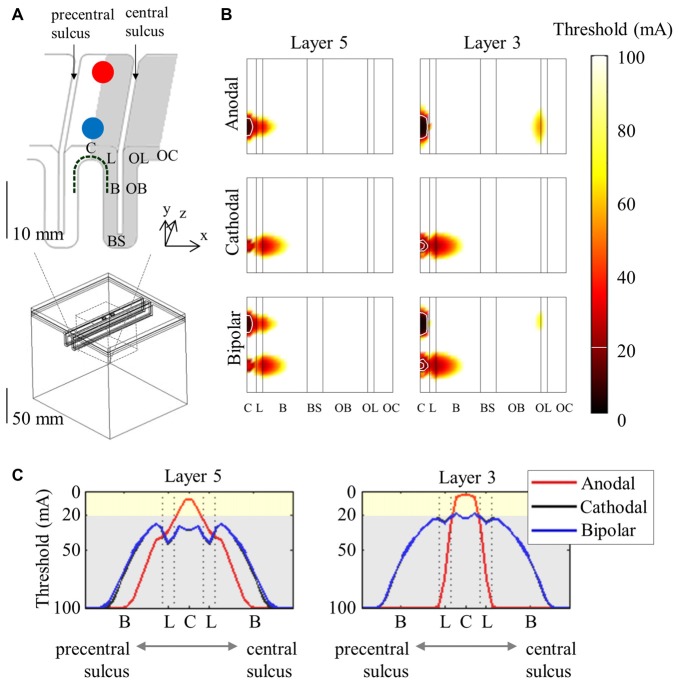
Spatial extent of excitation thresholds with various stimulus polarities (anodal, cathodal, and bipolar) in the simplified head model. A 3D view of the head model for subdural cortical stimulation **(A)** is shown; the gray matter is classified as the crown (C), lip (L), bank (B), the bottom sulcus (BS), which lies beneath the central sulcus and the opposite bank (OB), lip (OL), and crown (OC) located in the postcentral gyrus. The spatial extent of excitation thresholds **(B)** is visualized by stretching the gray matter surface (the gray colored region in **A**) in the *x*-direction. **(C)** We depict the excitation threshold along a dotted curve in **(A)**. Note that thresholds are symmetric because of the simple geometry of the model. Reproduced with permission from Seo et al. (2016a).

A more detailed morphology of PN obtained from cat visual cortex (Mainen and Sejnowski, 1996) was constructed with dimensions modified to fit the human motor cortex (Wongsarnpigoon and Grill, 2012; Seo et al., 2016a). Numerous neuronal models that cover the motor cortex were then modeled to determine target sites with varying stimulus parameters at the neuronal level. Figure 3 shows the spatial extent of the thresholds. Generally, neurons beneath the electrode had the lowest thresholds and the anodic threshold was lower than was that of the cathodic.

To resolve the uncertainties with respect to selective targeting stimulation protocols, Zwartjes et al. (2012) simulated axon models representing the intratelecenphalic type of basket cells, and pyramidal tract type cells depending on various stimulation parameters, such as montage (monopolar or bipolar stimulation), electrode polarity (anode or cathode), electrode orientation (parallel or perpendicular to the central sulcus), and electrode location (epidural or subdural). For example, they showed that cathodal stimulation stimulated basket cell axons selectively, and anodal stimulation showed the highest selectivity for PN’ axons.

## Modeling Activation of Cortical Neurons Produced by Noninvasive Brain Stimulation

TCS and TMS are noninvasive brain stimulation methods that represent electrical and magnetic stimulations. For electrical stimulation, the stimulus-induced potential field is calculated by solving the Laplace equation via the finite element method or boundary element method. Thereafter, an extracellular potential field is applied to neuronal models by extracellular stimulation and changes neuronal polarizations through voltage-gated ion channels (Stagg and Nitsche, 2011; Rahman et al., 2015). In contrast, magnetic stimulation induces an electric field,

(1) $$\vec{E}=-\partial \vec{A}/\partial t-\nabla \varphi$$

in which the magnetic vector potential ($\vec{A}$) is determined directly by the coil geometry, and the electric scalar potential (φ) is affected by charge accumulation at tissue interfaces. Thus, the electric field induced by magnetic stimulation was integrated into the neuronal models and transmembrane current was thus calculated using the time derivative of the electric field (Kamitani et al., 2001; Miranda et al., 2007; Pashut et al., 2011; Salvador et al., 2011; Rusu et al., 2014; Seo et al., 2016b). In one example (Pashut et al., 2011; Seo et al., 2016b), the external current was given by,

(2) $${\text{I}}_{\text{ext}}=-\frac{1}{{\text{r}}_{\text{a}}}\frac{\partial E_{l}}{\partial l},$$

where r_a_ is axial resistance, and *E*_l_ is the electric field that flows parallel (l) to each compartment of the neuronal models. Then, the external current was multiplied by waveforms that are time-derivatives of TMS coil current waveforms and this product stimulated neurons (Roth and Basser, 1990).

For magnetic stimulation, a simplified head model that is similar to the head model used in invasive stimulation was proposed with a stimulation coil (Silva et al., 2008; Salvador et al., 2011), and several neuronal models that consist of a single apical dendrite, soma, and axon were combined with the head model. The excitation thresholds depended on tissue heterogeneity, coil orientation (anterior-posterior or posterior-anterior), and pulse waveform (monophasic or biphasic). Tissue heterogeneity, which consists of changes in tissue conductivity, is an important factor in neuronal activation because it introduces a strong local gradient in the electric field at interfaces (Miranda et al., 2007). In addition, as the resulting electric field can influence the neuronal activations, changes in coil orientation influence neuronal excitability by inducing shifts in the spatial patterns of electric field changes (Opitz et al., 2013).

Rather than constructing a finite element head model, the stimulus-induced external currents that were generated by a magnetic coil were simulated by a RLC-circuit (Roth and Basser, 1990; Kamitani et al., 2001; Pashut et al., 2011), or were assumed to be a uniform electric field (Radman et al., 2009; Bikson et al., 2013; Rahman et al., 2013). For noninvasive stimulation, the uniform electric field is considered because of the small size of cortical neurons compared to the size of the stimulator, and weak electric stimulation (TCS) produces the approximately uniform electric fields seen in the head model. In the uniform electric field, compartments of neuronal models close to the anode are depolarized and those close to the cathode are hyperpolarized (Radman et al., 2009; Rahman et al., 2015). However, these simplified distributions of the electric field could potentially cause inaccurate predictions of neuronal responses by introducing certain potential modeling errors (McIntyre et al., 2004; Yousif et al., 2008; Ye and Steiger, 2015).

## High-Resolution Models for Brain Stimulation

The stimulus-induced electric field cannot be predicted easily because of the inhomogeneous properties and complex geometries of the cortex. To obtain precise information about the cortex, a high-resolution volume conductor head model (anatomically realistic head model) was constructed using MRI data (De Lucia et al., 2007; Datta et al., 2009, 2011; Lee et al., 2012; Edwards et al., 2013; Windhoff et al., 2013; Parazzini et al., 2014). These anatomically realistic head models help determine realistic electric fields that are undisputed in estimating the target area accurately.

**Figure 4:**
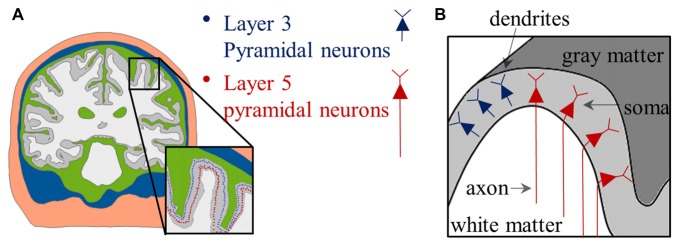
Placement of PN in the anatomically realistic head model. **(A)** The distributions of soma are indicated by colored dots. **(B)** Schematic view of the distribution of the PN; note that neurons have relative orientations according to their locations. Images modified with permission from Seo et al. (2016b).

**Figure 5:**
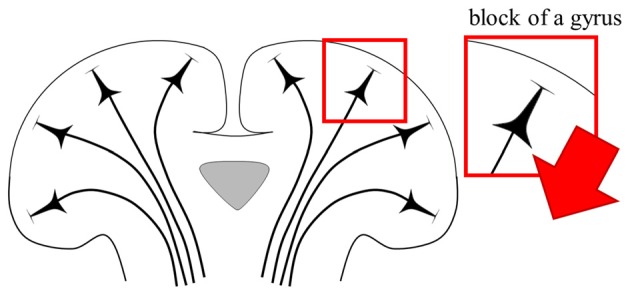
Schematic view of direction of PN. The red arrow illustrates the fixed direction of the axons of layer 5 PN. Note that the direction of the axons that runs straight is defined in a restricted block of a gyrus.

To perform a computational study of neural activations using the anatomically realistic head model, the distributions of the electric field are computed first. The next step is to combine neuronal models and the head model. Because of the irregular geometry of the anatomically realistic head model, all neuronal models have different orientations and therefore, positioning each is not straight forward. As Figure 4 shows, PN were oriented perpendicular to the cortical surface (DeFelipe et al., 2002; Manola et al., 2007; Wongsarnpigoon and Grill, 2012; Zwartjes et al., 2012; Seo et al., 2015, 2016b), and the axons for layer 5 PN were defined to bend in the direction of the internal capsule after they crossed the boundary between the gray and white matter. For example, we could locate neuronal models under the triangular element comprising the gray matter surface of the head model. As layer 3 PN were located within the cortex, their principal axis would align with the normal vector of a closed triangular surface element of the head model. Compared to layer 3 PN, modeling layer 5 PN is quite challenging because of their long axons. Thus, as shown in Figure 5, layer 5 PN were defined in a restricted area of the cortex (for example, a block of the precentral gyrus) that runs straight in the same direction. For the realistic axons of layer 5 PN, tractography using diffusion tensor imaging (DTI) data may be used (see “Neuromodulation in Tractography-Based White Matter Tracts” section).

Building multi-scale models using the anatomically realistic head model requires a significant amount of computation, manual work, and various imaging resources. However, although its use is complex, it may offer a better understanding of neurons’ responses. For example, when neuronal activations in the simplified head model were compared to those from the anatomically realistic head models, the latter provided a better understanding of layer 5 PN with asymmetric thresholds of spatial distributions (Seo et al., 2016b). In addition, the anatomically realistic head model allowed estimation of the anisotropic conductivity in white matter using DTI. When the influence of anisotropic white matter conductivity on the stimulation effects of invasive stimulation was investigated, anisotropic models showed a strong influence on layer 5 PN and observations consistent with the empirical findings that anodal stimulation has a lower threshold than does cathodal stimulation (Seo et al., 2015). Further, to achieve intense and focalized neuromodulation with minimal invasiveness, a transcranial channel that was implanted in the patient’s skull was introduced (Wingeier and Pless, 2015), and thereafter, the channel’s effect on activation of cortical neurons was investigated using the anatomically realistic head model (Seo et al., 2017a,b). The transcranial channel combined with TCS with direct current (transcranial direct current stimulation: tDCS) was introduced, and head models with two types of tDCS montages, a conventional tDCS using large patch-type electrodes and high-definition tDCS (HD-tDCS) using small disc-type electrodes, were constructed with and without the transcranial channel. Seo et al. (2017a,b) found that the transcranial channel allowed a better targeting neuromodulation that increased both spatial focality and intensity of the neuronal excitability at the target area.

**Figure 6:**
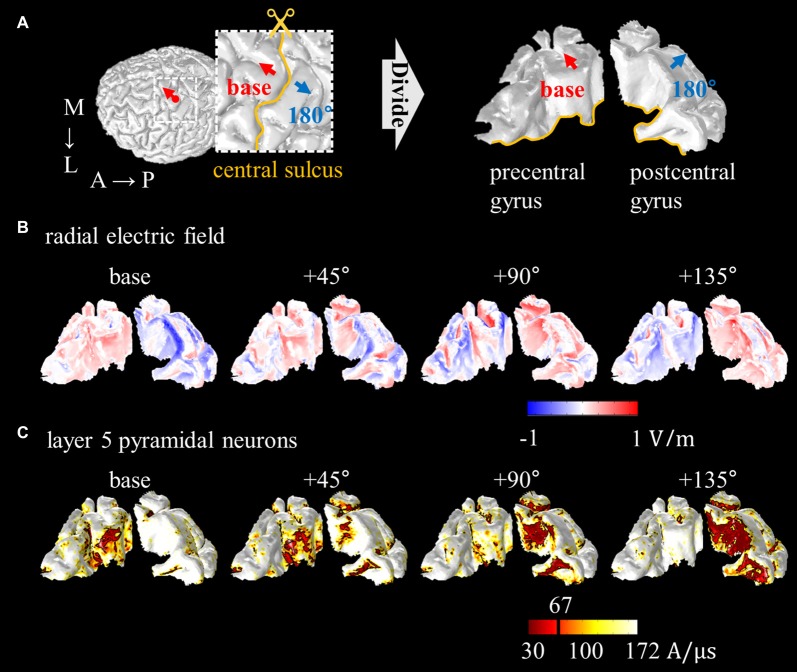
Spatial extent of radial electric field and threshold distribution for layer 5 PN as a function of coil orientations. **(A)** The precentral and postcentral gyrus are divided for visualization purposes. The excitability predicted based on the radial electric field **(B)** and excitation threshold for layer 5 PN **(C)** are illustrated. Images modified with permission from Seo et al. (2016b).

Some attempts have been made to explain neuronal excitability using the anatomically realistic head model for magnetic stimulation. Goodwin and Butson (2015) proposed multi-scale models for TMS by integrating the anatomically realistic head model with layer 3 PN, and found a strong correlation between coil orientation and excitation threshold. Seo et al. (2016b) reported consistent observations, and thus, the spatial extent of thresholds was characterized as a function of coil orientation, as shown in Figure 6. In addition, activation sites showed matching predictions based on the radial electric field that flows perpendicular to the cortical surface, with active areas in the sulcal walls, because the radial field flows parallel to the somatodendritic axis of PN. It may be possible to use the radial electric field as a simple way to predict areas of neuronal activation, and neuronal models permit a more detailed understanding of the biophysical mechanisms.

## Neuromodulation in Tractography-Based White Matter Tracts

Modeling realistic axons can be performed by tractography using DTI; eigenvectors derived from such imaging represent axon directions within the white matter (Jones and Leemans, 2011), while axons within the gray matter are assumed to be oriented perpendicular to the cortical surface. Further, the quality of tractography (for example the smooth bent part) is improved when a custom algorithm implemented in Matlab is used (Nummenmaa et al., 2014; Shahid et al., 2014).

To achieve a better understanding of the effect of the electric field on neuronal excitability, the spatial gradient of the electric field (the activating function) along the tractography-based fibers (axons) was estimated for noninvasive stimulation (Opitz et al., 2011; Nummenmaa et al., 2014; Shahid et al., 2014; De Geeter et al., 2015). For magnetic stimulation, Opitz et al. (2011) reconstructed several fibers, Nummenmaa et al. (2014) extended fiber models to include a block of gyrus, and De Geeter et al. (2015) constructed substantially larger fibers; fibers showed maximal activations when they were aligned with the direction of the electric field. Shahid et al. (2014) reported neural excitability for tDCS that was highly sensitive to the direction of the electric field with respect to the fiber path. De Geeter et al. (2016) proposed further modeling approaches that compartmentalize tractography-based fibers into single dendrities, soma, axon hillock, initial segment, and myelinated axons, and by solving the cable equation to predict membrane polarizations, they showed the way in which TMS modulates neuronal excitability beyond the superficial area. While DTI-based tractography provides information about axonal architectures, fibers are reconstructed by the bulk-averaged properties and are contaminated by noise that reduces precision (Tournier et al., 2011; Jeurissen et al., 2013). Further, as simulation of neural stimulation using tractography-based tracts has focused on the activating function along the tracts or simplified neuronal models, future work should investigate realistic morphology and various types of neurons (De Geeter et al., 2016).

## Implications for Future Modeling Work

While considerable literature has investigated brain stimulation effects using the magnitude of the stimulus-induced electric field, multi-scale models demonstrated that the spatial extent of excitation thresholds was not consistent with the distributions of the electric field. For example, an electric field with a higher magnitude induced by magnetic stimulation was focused on the top of the gyrus, while neural activations were observed in the sulcal walls (Janssen et al., 2015; Seo et al., 2016b). In contrast, the radial electric field showed matching target areas that resulted from PN, which might be attributable to these neurons’ orientation parallel to the radial field (Rushton, 1927; Fox et al., 2004; Krieg et al., 2013). Although electric field orientation might be a major factor in determining neural targets, it cannot reflect variable neural responses attributable to different morphologies and electrical properties of cortical neurons. Multi-scale modeling may provide detailed responses of neurons, such as the initiation sites of action potentials and precise target areas. In addition, when multi-scale models are constructed using the anatomically realistic head model, we can construct individualized models and observe the variation of neural responses across subjects.

There are challenges in the technical features of multi-scale modeling, which entails a two-step process:

- First, the electric field distribution produced by brain stimulation is computed using the head models.
- Second, multi-compartmental models of cortical neurons are constructed virtually in the head models, and then external electric fields are applied to the neuronal models.

Two types of head models, simplified and anatomically realistic, are used in the multi-scale models. The simplified head model is highly efficient with respect to computational time and it is easy to couple neuronal models with the head model directly. Because of the speed of the simplified head model, spherical models comprising concentric spheres are used widely in present navigators for TMS. However, they may provide inaccurate patterns of the stimulus-induced electric field that result in falsely guided stimulation (Nummenmaa et al., 2013). The importance of the anatomically realistic head model in realistic electric field calculation has been recognized, as it models precisely both stimulation parameters and the relevant anatomy (Bikson et al., 2012; Windhoff et al., 2013). In addition, the model can apply anisotropic conductivity derived from DTI to the white matter. One of the free software programs used to simulate noninvasive stimulation is SimNIBS, which provides a script for generation of the anatomically realistic head model using MRI (Thielscher et al., 2015). SimNIBS integrates various free software packages in FreeSurfer (Dale et al., 1999), FSL (Jenkinson et al., 2012), Gmsh (Geuzaine and Remacle, 2009), meshfix (Attene and Falcidieno, 2006), and GepDP (Dular et al., 1998) for segmentation of MRI, meshing and finite element calculations, and the generation of the head model takes up to 15 h according to the pipeline SimNIBS. Thus, although the anatomically realistic head model provides more accurate insight into electric field patterns, the procedure required to generate the model is very time consuming. Despite these computational costs, the anatomically realistic head model is used because of its merit, in that it may help extrapolate individual physiological and therapeutic effects that vary substantially.

A further challenge arises in coupling the neuronal and the head model, which has a complex and diverse geometry. Although the anatomically realistic head model requires intricate procedures to combine neuronal models because of this geometry, recent analyses have focused on it because it may provide more precise predictions at the macroscopic and microscopic levels. For typical PN, it is intuitive to couple layer 2/3 neurons with the head model by aligning them perpendicular to the cortex in the same way that layer 2/3 neurons are positioned within the gray matter. In contrast, modeling layer 5 PN is quite complicated because their axons extend into the white matter. When layer 5 neurons are combined with the simplified head model, the axons extend straight because of their simple geometry. In the anatomically realistic head model, the direction of the axons might be fixed in a specific direction by restricting neuronal distributions to a small area (for example, a block of the precentral gyrus; see Figure 5) or be extrapolated using the eigenvectors acquired from DTI. A recent multi-scale model proposed using tractography to couple the anatomically realistic head model with multi-compartmental neuronal models that consist of single dendrites, soma, and myelinated axons (De Geeter et al., 2016). In modeling studies, thresholds for the direct responses and spikes for indirect waves varied greatly depending on dendrite morphology (Wongsarnpigoon and Grill, 2012; Rusu et al., 2014). In addition, the inclusion of axon collaterals induced changes in the activation of PN, while other models without collaterals showed inactivation (Zwartjes et al., 2012). Therefore, future multi-scale models that apply detailed compartmental models of neurons and tractography-based fibers may be promising, as such detailed morphology may improve the prediction of neuronal responses.

Patton and Amassian (1954) were the first to describe an initial positive deflection, D-wave (direct response), followed by an I-wave (indirect response), which is a series of variable positive waves that follow synaptic excitation at longer latencies. At the minimal stimulus threshold and amplitude required to evoke neuronal activation, anodal stimulation usually elicited the D-wave, while cathodal stimulation typically evoked the I-wave. With a supra-threshold stimulus, D- and I-waves were observed with both anodal and cathodal stimulation (Gorman, 1966). Currently, most studies that have used multi-scale models to measure neuronal excitability have constructed isolated neuronal models, and thus, these works predicted the D-wave by direct stimulation of neurons. This direct response of single PN is invaluable, because PN are known to be the primary activators of the corticospinal tract that issues from the cortex, and direct activation may provide evidence of cells that may be stimulated synaptically (Phillips and Porter, 1962; Gorman, 1966).

Hern et al. (1962) reported comparable muscle motor responses following anodal and cathodal stimulations, while the D-wave had a generally lower threshold in anodal stimulation compared to that in cathodal stimulation. In addition, when electrical stimulation was applied to awake subjects through contacts placed chronically on the motor cortex, cathodal stimulation induced neural responses more readily than did anodal stimulation (Hanajima et al., 2002). These results implied that neurons largely were activated indirectly under certain conditions. However, the question of which neurons are activated precisely to induce the I-wave, and the underlying mechanism, remains unanswered. To obtain some insight about the I-wave, circuit models were used to estimate the activation of D-and I-waves. For example, Esser et al. (2005) constructed a large-scale model for a thalamocortical circuit that was composed of single-compartment excitatory and inhibitory neurons, and Rusu et al. (2014) made a pool of layer 2/3 neurons and a detailed layer 5 pyramidal neuron with synaptic connections. As with the circuit model, they reproduced well responses recorded epidurally to TMS, especially the I-wave, but did not consider magnetic fields explicitly. Future modeling approaches that combine head and circuit models may provide deeper insight into the underlying mechanism of D- and I-waves with myriad combinations of stimulation settings (for example, coil/electrode position, amplitude, and stimulus frequency).

These modeling studies presented lack explicit validation, and modeling results are confirmed commonly by established experimental observations. In electrical stimulation, neurons located perpendicular to the electrode showed depolarization in the axon and hyperpolarization in apical dendrites during anodal stimulation, and concomitantly, the opposite polarization patterns were observed in cathodal stimulation (Hern et al., 1962; Gorman, 1966). Consistent patterns of neuronal polarization according to stimulus polarities have been found in modeling results of electrical stimulation. For example, tDCS with an active electrode (anode) placed close to the precentral gyrus activated neurons in compartmental-specific manners; apical dendrites were hyperpolarized, and compartments below the soma, including basilar dendrites, were depolarized simultaneously (see Figure 3 in Seo et al., 2017b). For magnetic stimulation, while the TMS-induced electric field is constrained on the top of the gyrus, cortical activation in the sulcus has been observed via imaging and modeling studies (Fox et al., 2004; Krieg et al., 2013; Janssen et al., 2015; Seo et al., 2016b). Further, physiological experiments have shown that the largest amplitude of motor evoked potential is elicited when currents flow posterior to anterior (Mills et al., 1992; Balslev et al., 2007). Consistently, multi-scale models for magnetic stimulation have shown that larger areas in the sulcal wall along the central sulcus, which is indicated by the hand knob (Yousry et al., 1997), were targeted when the TMS coil was oriented 45° to the midline (Goodwin and Butson, 2015; Seo et al., 2016b). Although modeling studies have found some observations consistent with experimental studies, validation remains a difficult challenge because of the uncertainty of many factors that affect the outcomes of neuromodulation, for example, the tissue properties of head models (Bikson et al., 2012; Lee et al., 2012), morphology and electrical properties of neuronal models (Wongsarnpigoon and Grill, 2012), and variability in the population (Edwards et al., 2013). In particular, precise parameters of neuronal models are essential to explore the mechanism of cellular activity, but in the absence of the electrical and geometrical properties of human cortical neurons, multi-scale models incorporate simplified neuronal models (Manola et al., 2005, 2007; Salvador et al., 2011; De Geeter et al., 2015, 2016) or are based on cells from animal cortices (Mainen and Sejnowski, 1996; Schaefer et al., 2003; Larkum et al., 2009). Further, the morphologies of neurons vary among species (DeFelipe et al., 2002), and changes in the membrane properties and ion channels have the greatest influence in predicting excitation thresholds. Thus, future studies should focus on the uncertainty of neurons’ properties and parametric analyses to determine which parameters might contribute to neuromodulation of brain stimulation.

## Conclusions

Computational studies using volume conductor head models have demonstrated a stimulus-induced electric field that interpolates the target area activated, and thus cannot deduce detailed neuronal responses. Numerical studies using a multi-compartmental model of neurons have shown computed neural responses, but they cannot provide extensive target sites and have little control over external stimulation parameters. To guide cellular targets induced by brain stimulation with myriad combinations of stimulus parameters, recent literature has introduced multi-scale models that combine volume conductor head models and multi-compartmental neuron models. In addition, because it is necessary to estimate realistic electric fields using the anatomically realistic head model, constructing multi-scale models with this model is an undisputed method that provides accurate activated cellular targets. Thus, it may be important to increase the realism of multi-scale models further. Therefore, future studies may illustrate the cellular responses in a more detailed manner and further our understanding of the underlying mechanisms during brain stimulation.

## Author Contributions

HS performed the literature survey, and wrote the article. SCJ oversaw all survey procedures and approved the final manuscript.

## Conflict of Interest Statement

The authors declare that the research was conducted in the absence of any commercial or financial relationships that could be construed as a potential conflict of interest.
